# Unraveling Visual Field Asymmetry: Insights Into Left-Right Differences in Glaucoma Patients

**DOI:** 10.7759/cureus.79711

**Published:** 2025-02-26

**Authors:** Fumio Takano, Sotaro Mori, Iwaki LNU, Mina Okuda-Arai, Kaori Ueda, Mari Sakamoto, Yuko Yamada-Nakanishi, Makoto Nakamura

**Affiliations:** 1 Division of Ophthalmology, Department of Surgery, Kobe University Graduate School of Medicine, Kobe, JPN

**Keywords:** angle-closure mechanism, glaucoma disease type, left-right difference, mixed glaucoma, primary open angle glaucoma

## Abstract

Purpose: Primary open-angle glaucoma (POAG) typically exhibits bilateral symmetry in visual field defects, while secondary glaucoma often manifests substantial left-right differences. This study investigates the diagnostic relevance of left-right differences in the Humphrey visual field (HVF) test and explores the factors influencing these differences.

Study design: This is a cross-sectional study.

Methods: Parameters were assessed in 201 glaucoma patients, including age, sex, glaucoma disease type, central corneal thickness (CCT), corneal endothelial cell density (ECD), axial length, anterior chamber depth, refractive power, intraocular pressure (IOP), glaucoma drug score, and mean deviation (MD), pattern standard deviation (PSD), and visual field index (VFI) for both eyes in HVF. Patients were categorized into type 1 (POAG in both eyes) and type 2 (secondary glaucoma). Multivariable analysis was conducted to explore factors influencing left-right visual field test differences.

Results: No significant differences were found between type 1 and type 2 in left-right MD, PSD, and VFI (p=0.13, 0.26, 0.09). Type 2 exhibited significant inter-eye differences in ECD, CCT, IOP, and glaucoma drug scores (p=0.02, <0.01, <0.001, 0.01). In the type 1 group, the left and right MD values were statistically significantly correlated (r=0.40, p<0.000001), but 24.6% of patients showed a left-right difference of 10 dB or more. Multivariable regression analysis identified anterior chamber depth as the sole significant factor influencing left-right MD differences in POAG (p=0.03).

Conclusion: Asymmetry in the visual field cannot distinguish between glaucoma disease types. In POAG, a shorter anterior chamber depth is associated with increased left-right MD differences, emphasizing its significance in understanding the progression of visual field defects.

## Introduction

Within the realm of glaucoma treatment, accurate identification of the specific glaucoma disease type is crucial. For instance, exfoliation glaucoma progresses approximately twice as fast as primary open-angle glaucoma (POAG), with a mean deviation (MD) change of -0.64 ± 0.7 dB/year compared to -0.35 ± 0.3 dB/year (p < 0.01) [1]. Patients with uveitic glaucoma, exemplified by cytomegalovirus-positive Posner-Schlossmann syndrome, experience a substantial MD reduction of -2.6 ± 2.4 dB/year, indicating secondary glaucoma associated with rapid visual field defects [2]. Vigilant recognition of these high-risk subtypes is essential to guide timely and appropriate treatment strategies.

Conversely, clinicians may encounter difficulty in identifying the secondary nature of their condition in patients with secondary glaucoma. While exfoliation glaucoma is typically diagnosed through the deposition of white fibrous material on the lens or pupil margins, this might go unnoticed, particularly in patients who have previously undergone cataract surgery at another facility.

While POAG typically exhibits a uniform degree of visual field defect in both eyes [3], secondary glaucoma often manifests unilaterally. For example, a study on exfoliation syndrome found it to be slightly more common unilaterally than bilaterally when clinically detected [4]. Another study reported that uveitic glaucoma caused by the herpes virus is typically unilateral [5]. However, no literature to date has demonstrated that a distinct difference between left and right visual field tests serves as an indicator of secondary glaucoma. This study aims to investigate whether a significant asymmetry in visual field defects between the left and right eyes could provide useful clinical insights for identifying secondary glaucoma.

## Materials and methods

This cross-sectional study targeted patients scheduled to visit our glaucoma outpatient clinic at Kobe University Hospital between 01 Jan 2023 and 31 Dec 2023. Inclusion criteria for both eyes aligned with the Humphrey visual field test 30-2 Swedish Interactive Thresholding Algorithm (SITA)-Standard. Patients with concurrent eye or brain diseases, aside from glaucoma, were excluded. However, individuals with mild cataracts or superficial punctate keratitis deemed unlikely to impact visual field test results were included. Patients who had undergone glaucoma surgery in the past or cataract surgery within one year of data collection were excluded. This study adhered to the tenets of the Declaration of Helsinki and was approved by the Institutional Review Board of Kobe University (No. B230207).

The collected data encompassed various parameters, including age, sex, glaucoma disease type, central corneal thickness, corneal endothelial cell density, axial length, anterior chamber depth, intraocular pressure (IOP), glaucoma drug score, and MD/pattern standard deviation (PSD)/visual field index as values derived from the Humphrey visual field test for both left and right eyes. Data with false positives of 15% or more and false negatives of 33% or more were excluded.

Glaucoma diagnosis adhered to Anderson Patella's criteria [6]. Glaucoma specialists (SM, KU, MS, YY-N, MN) categorized glaucoma types into six groups: normal, POAG including normal tension glaucoma, exfoliation glaucoma, steroid-induced glaucoma, primary angle-closure glaucoma, and other secondary glaucoma such as uveitis or trauma-induced. The determination of open or closed angle was made at the initial examination using a gonioscope, defining it as an anatomical angle malposition of 270 degrees or more, not obstructed by the iris [7].

Measurements of axial length and anterior chamber depth were conducted using an IOLMaster® (Carl Zeiss Meditec; Dublin, CA, USA), central corneal thickness with a Noncon. Robo® (Konan Medical, Nishinomiya, Japan), and IOP with Goldmann applanation tonometry. These parameters were recorded within three months before or after the visual field test. The glaucoma drug score, as previously reported, assigned 1 point for single drug administration and 2 points for combination drug eye drops [8]. No patients received oral carbonic anhydrase inhibitor administration and combination eye drops, which contain three or more agents.

We categorized type 1 as the bilateral POAG group and type 2 as patients with secondary glaucoma in one or both eyes. We examined whether there were differences in the laterality of each parameter between type 1 and type 2. To explore the factors influencing the left-right difference in MD of Humphrey visual field testing, multivariable analysis incorporated the mentioned parameters. The objective variable was the left-right difference in MD value, with explanatory variables including age, sex, axial length, anterior chamber depth, and left-right difference in anterior chamber depth, axial length, corneal endothelial cell density, and IOP. The shorter value for both eyes was utilized for axial length and anterior chamber depth.

## Results

Table 1 provides a comprehensive summary of data for both eyes and left-right differences among the 201 glaucoma patients, including the differences between the left and right eyes. Discrepancies in patient numbers between left and right disease types arose from cases where different disease types were identified on the left and right sides. Neovascular glaucoma cases were excluded based on criteria excluding other eye diseases.

**Table 1:** Patient demographics POAG: Primary open-angle glaucoma, IOP: Intraocular pressure, HVF: Humphrey visual field. Continuous variables were shown as medians (interquartile range), and categorical variables were shown as numbers (proportion).

Items (n=201)	Right Eye	Left Eye	Left-Right Difference
Sex, male/female (male ratio)	107/104 (53.2)		
Age, y.o.	66 (54, 73)		
Glaucoma Disease Type			
Normal	11 (5.5)	8 (4.0)	
POAG	135 (67.2)	138 (68.7)	
Exfoliation Glaucoma	22 (10.9)	18 (9.0)	
Steroid-Induced Glaucoma	7 (3.5)	8 (4.0)	
Primary Angle Closure Glaucoma	7 (3.5)	7 (3.5)	
Other Secondary Glaucoma	19 (9.5)	22 (10.9)	
Central Corneal Thickness, μm	527 (497, 555)	524 (496.5, 552.8)	14 (6, 29)
Endothelial Corneal Cell Density, cells/mm^2^	2655 (2410.8, 2840.5)	2688.5 (2416.3, 2864)	129 (60, 236)
Axial Length, mm	25.31 (23.97, 26.80)	25.21 (23.91, 26.91)	0.20 (0.09, 0.36)
Anterior Chamber Depth, mm	3.51 (3.15, 3.89)	3.53 (3.20, 3.98)	0.12 (0.05, 0.34)
IOP, mmHg	15 (13, 18)	15 (13, 19)	2 (1, 4)
Glaucoma Drug Score	3 (1, 4)	3 (1, 4)	0 (0, 1)
Mean Deviation of HVF, dB	-10.26 (-4.30, -16.72)	-9.08 (-4.77, -16.68)	5.25 (2.64, 11.02)
Pattern Standard Deviation of HVF, dB	10.62 (4.23, 13.29)	9.80 (5.73, 12.99)	3.28 (1.13, 6.60)
Visual Field Index of HVF, %	74 (55.5, 93)	77 (51, 89)	15 (4, 33)

**Figure 1:**
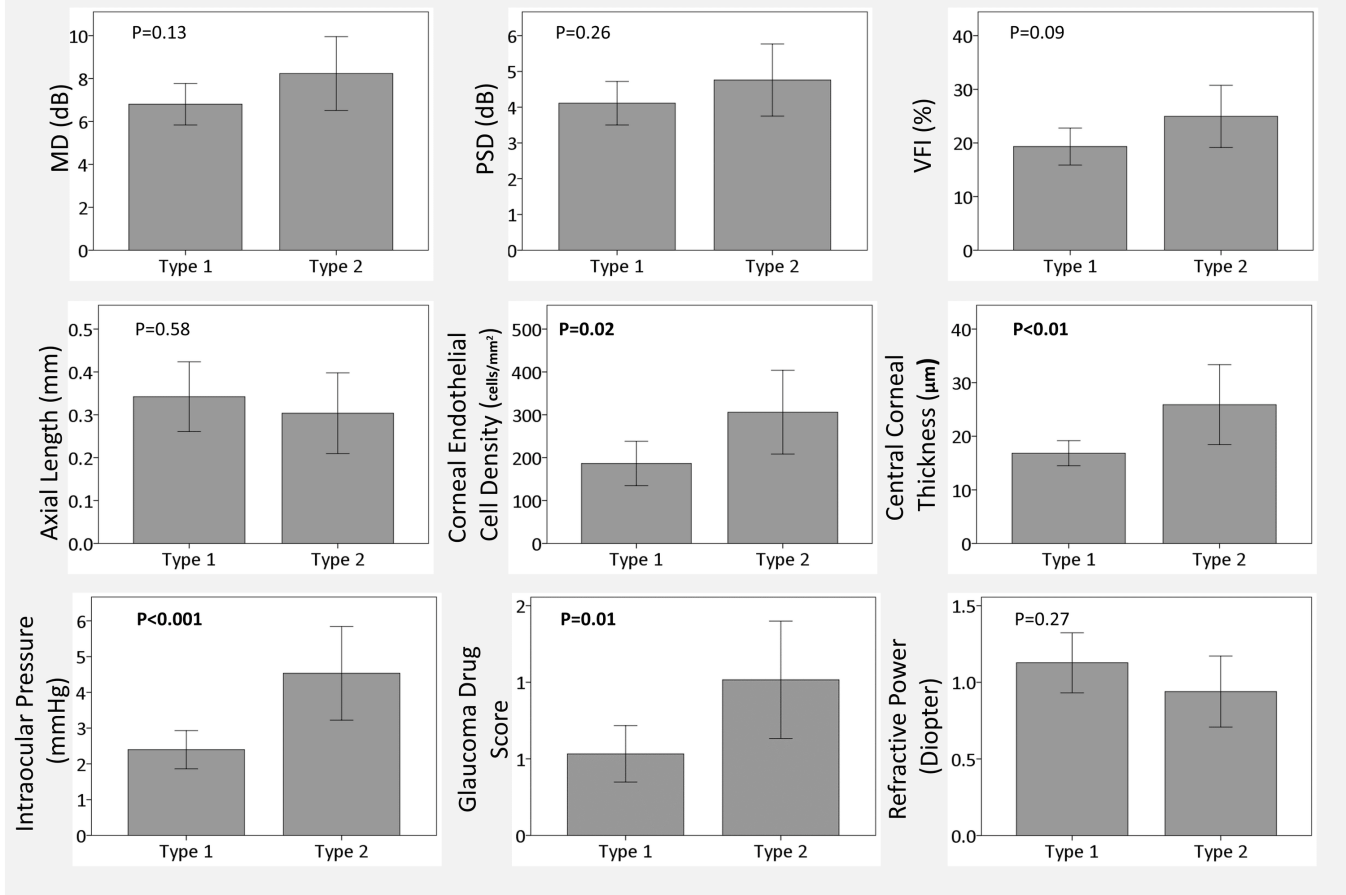
Left-right difference of each parameter Type 1 indicates primary open-angle glaucoma in both eyes, and type 2 indicates secondary glaucoma in one or both eyes. P-values indicate the results of the Mann-Whitney test. P-values in bold indicate statistically significant.

Figure 1 compares differences between two groups for each parameter, with type 1 representing a POAG group in both eyes and type 2 representing patients with secondary glaucoma in one or both eyes. Contrary to expectations, no significant differences were observed between the two groups in visual field test values such as MD, PSD, and VFI (p=0.13, 0.26, 0.09, respectively). However, the type 2 secondary glaucoma group exhibited significantly larger left-right differences in corneal endothelial cell density and central corneal thickness (p= 0.02, <0.01). Additionally, left-right differences in IOP and glaucoma drug scores were larger in type 2 than in type 1 (p<0.001, 0.01).

**Figure 2:**
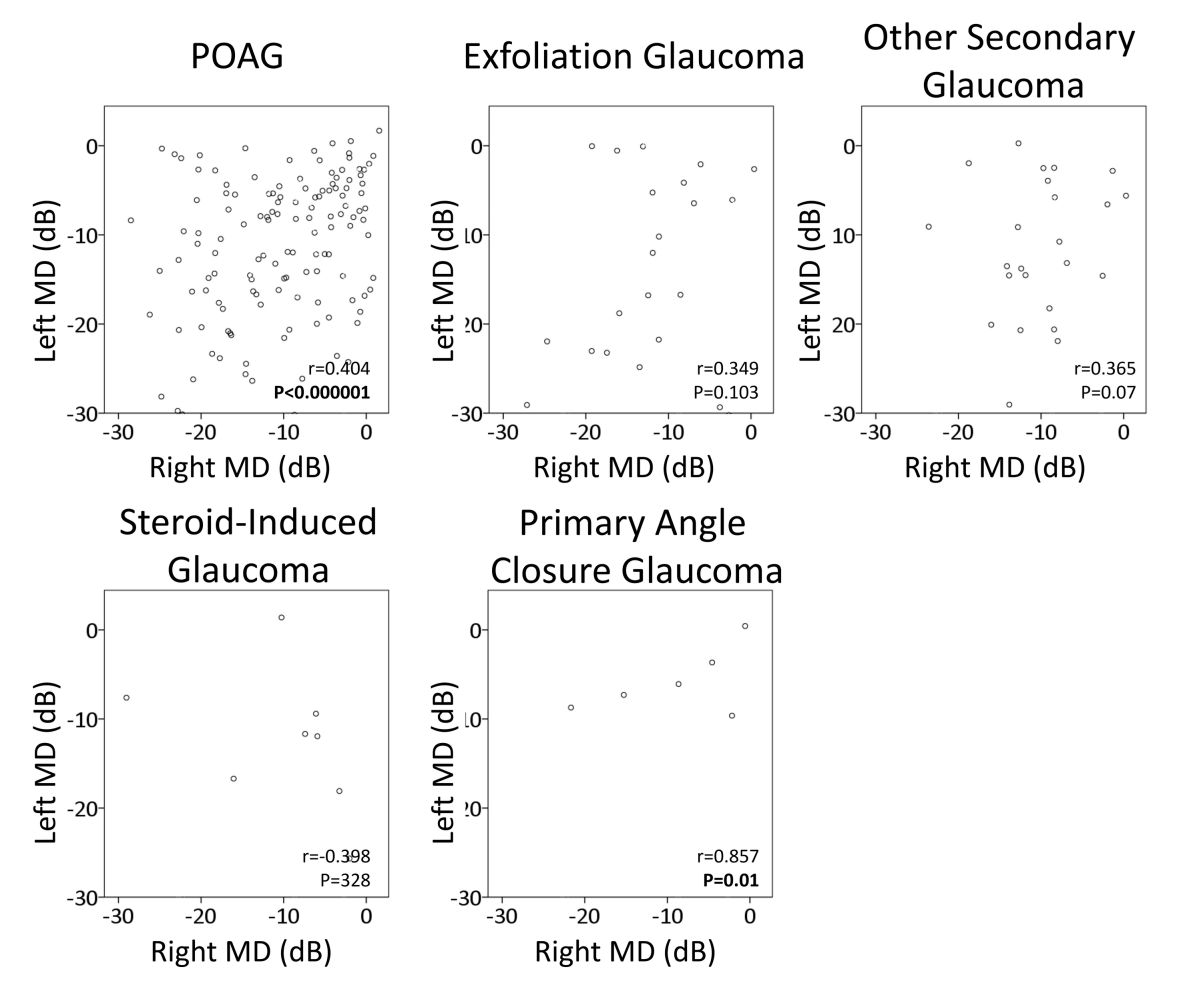
Scatter plot of left and right MD value for each disease type MD: Mean Deviation of Humphrey Visual Field Test. POAG: Primary open-angle glaucoma. R and p-values indicate Pearson product-moment correlation coefficient, and significant ones are shown in bold.

Figure 2 depicts the left and right MD values in five groups: POAG, exfoliation glaucoma, other secondary glaucoma, steroid-induced glaucoma, and primary angle-closure glaucoma. In the POAG group, a significant correlation between left and right MD values was observed (r=0.40, p<0.00001), although some cases showed a substantial difference of -10 dB or more (24.6%). Among other glaucoma groups, the angle-closure glaucoma group exhibited a significant correlation (p=0.01), but no significant correlation was noted between the left and right sides in other groups.

**Figure 3:**
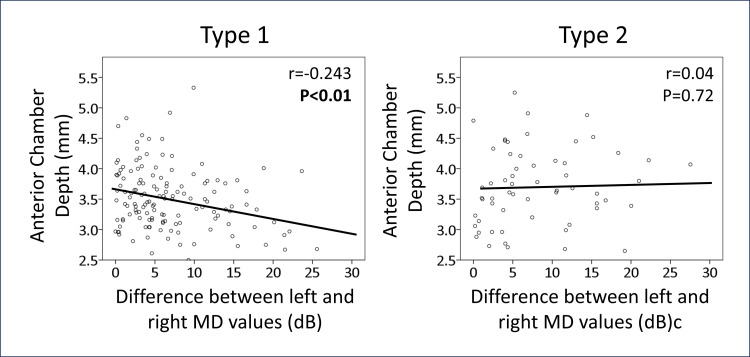
Relationship between left-right difference in MD and anterior chamber depth MD: Mean deviation of Humphrey visual field test. Type 1 indicates primary open-angle glaucoma in both eyes, and type 2 indicates secondary glaucoma in one or both eyes. R and p-values indicate Pearson product-moment correlation coefficient, and significant ones are shown in bold.

Surprisingly, a left-right difference in MD values was noted in patients with POAG, prompting multivariable regression analysis, as detailed in Table 2. The results indicated that anterior chamber depth was a significant factor (dds ratio: -2.32, p=0.03), with a shallower anterior chamber depth correlating with a greater left-right difference in MD values. Figure 3 further illustrates this correlation, revealing a significant association in the bilateral POAG group (r=-0.243, p<0.01), while no such trend was observed in the secondary group (r=0.046, p= 0.72).

**Table 2:** Multivariable regression analysis of factors determining the differences between left and right MD values of the Humphrey visual field test in POAG patients POAG: Primary open-angle glaucoma, ACD: Anterior chamber depth, IOP: Intraocular pressure, ECD: Endothelial corneal cell density. P-values in bold indicate statistically significant.

Items (n=134)	β (95% CI)	P-value
Age, year	0.03 (-0.05, 0.11)	0.26
Sex to Male	2.43 (-0.44, 4.43)	0.18
Axial Length, mm	-0.20 (-0.94, 0.53)	0.58
Lateral Difference in Axial Length, mm	0.34 (-1.88, 2.54)	0.76
ACD of the shorter eye, mm	-2.32 (-4.47, -0.16)	0.03
Lateral Difference in ACD, mm	1.76 (-1.30, 4.82)	0.26
Lateral Difference in IOP, mmHg	0.19 (-0.11, 0.50)	0.22
Lateral Difference in ECD, cells/mm^2^	0.00 (-0.01, 0.01)	0.81

## Discussion

In this study, we observed that left-right differences in visual field test parameters do not serve as a distinguishing factor between POAG and secondary glaucoma. Surprisingly, some cases of POAG exhibited substantial left-right differences in MD values, and anterior chamber depth emerged as a significant factor influencing these differences in POAG patients.

Previous studies have established a correlation between the degree and pattern of visual field defects in both eyes of individuals with POAG [3]. Conversely, studies on normal-tension glaucoma often reveal asymmetry in visual field defects, with conflicting opinions on whether these disparities are related to IOP [9] or not [10-12]. Moreover, in untreated normal-tension glaucoma, the mean blur rate in laser speckle flowgraphy is reported to be more correlated than IOP or ocular perfusion pressure [13], raising questions about the role of IOP in glaucoma progression.

Our study found that left and right corneal endothelial cell density, central corneal thickness, IOP, and glaucoma drug score were greater in the secondary glaucoma group than in the POAG group. Exfoliation syndrome and cytomegalovirus (CMV) infection, known to decrease corneal endothelium, influenced the significant differences in corneal endothelial cell density values in the secondary glaucoma group. Compared to POAG, secondary glaucoma can cause explosive increases in IOP, and such a secondary glaucoma nature may have increased the left-right difference in IOP and glaucoma drug score in the secondary glaucoma group. Corneal thickness differences may be attributed to certain glaucoma eye drops [14,15] and corneal epithelial edema with elevated IOP.

Additionally, we identified a new factor, anterior chamber depth, as a determinant of left-right differences in visual field defects in POAG patients. This finding suggests the involvement of an angle-closure mechanism in POAG, challenging the traditional separation of open-angle and angle-closure glaucomas. A pathological condition that has both open-angle and closed-angle is sometimes described as mixed mechanism glaucoma. However, a past article pointed out that this "mixed mechanism" is a rare situation, and we must avoid using such a term [16]. On the other hand, it has been shown that short axial length or hyperopic eyes are more likely to develop visual field defects, even in open-angle glaucoma [17,18]. In other words, these previous reports suggest that the angle-closure mechanism is also at work in open-angle glaucoma.

The progression of visual field defects in individuals with narrow anterior chamber depths, even in the presence of an apparently open angle, may be explained by the larger fluctuations in IOP observed with shorter axial lengths [19,20]. There is a possibility that elevated IOP may go unnoticed in the medical examination. Due to such unnoticed changes in IOP, visual field defects rapidly progress in eyes with shallow anterior chambers, leading to large left-right differences.

The hypothesis that angle-closure glaucoma coexists with open-angle glaucoma also gains support from glaucoma surgery results. Minimally invasive glaucoma surgery (MIGS), particularly Schlemm's canal surgery, demonstrates improved outcomes when combined with concomitant cataract surgery [21-23], emphasizing the potential role of the angle-closure mechanism in primary open-angle glaucoma.

Our limitation stems from the heterogeneity of our patient cohort, which included individuals with both phakic and pseudophakic eyes, as well as various presentations of secondary glaucoma. We analyzed only IOL eyes, but no significant relationship was observed. This is probably due to the reduced number of cases and the inclusion of cases in which the angle-closure mechanism had progressed and the difference between the left and right eyes had expanded before cataract surgery, and the condition stabilized after surgery and follow-up at our hospital. While patients with glaucoma were categorized into POAG and other secondary glaucomas, it appears that the likelihood of left-right differences varies depending on the specific type of secondary glaucoma. Interestingly, despite this variability, we observed a significant correlation between anterior chamber depth and left-right differences in MD values. The discovery of the angle-closure factor associated with POAG was serendipitous in this study, highlighting the need for further validation with this aspect as the primary outcome. Although we observed a relationship between anterior chamber depth and left-right differences in MD values, we did not find a correlation between left-right differences in anterior chamber depth. We hypothesize that patients with a pronounced angle-closure glaucoma mechanism may experience accelerated visual field narrowing, leading to more pronounced left-right differences. In this study, the agreement between the left and right eyes is represented by the correlation coefficient in Figure 2. However, this calculation method depends on the sample size, and the value may vary for other disease types.

## Conclusions

Our investigation into the laterality of eyes in glaucoma patients revealed unexpected left-right differences in visual field defects, notably in patients with POAG. The short anterior chamber depth emerged as a significant factor contributing to these differences. Our findings suggest that even when diagnosed with primary open-angle glaucoma, an angle-closure mechanism may be present when bilateral differences in visual field defects manifest in both eyes.
